# The effect of high-fructose corn syrup vs. sucrose on anthropometric and metabolic parameters: A systematic review and meta-analysis

**DOI:** 10.3389/fnut.2022.1013310

**Published:** 2022-09-27

**Authors:** Xiang Li, Yunqi Luan, Yuejin Li, Shili Ye, Guihui Wang, Xinlun Cai, Yucai Liang, Hamed Kord Varkaneh, Yunpeng Luan

**Affiliations:** ^1^The First Affiliated Hospital of Yunnan University of Traditional Chinese Medicine, Kunming, China; ^2^Beijing Institute for Drug Control (Beijing Center for Vaccine Control), Beijing, China; ^3^The General Surgery Department, The First People's Hospital of Yunnan Province, Kunming, China; ^4^Faculty of Mathematics and Physics, Southwest Forestry University, Kunming, China; ^5^Department of Endocrinology, Kunming Municipal Hospital of Traditional Chinese Medicine, Kunming, China; ^6^Lairui Biotechnology (Yunnan) Co., Ltd. Yunnan, China; ^7^Key Laboratory for Forest Resources Conservation and Utilization in the Southwest Mountains of China, Ministry of Education, Southwest Forestry University, Kunming, China

**Keywords:** high-fructose corn syrup, sucrose, fructose, weight, meta-analysis

## Abstract

High-fructose corn syrup (HFCS) has been speculated to have stronger negative metabolic effects than sucrose. However, given the current equivocality in the field, the aim of the present study was to determine the impact of HFCS use compared to sucrose on anthropometric and metabolic parameters. We searched PubMed, Scopus, Cochrane Central and web of sciences, from database inception to May 2022. A random effects model and the generic inverse variance method were applied to assess the overall effect size. Heterogeneity analysis was performed using the Cochran Q test and the I^2^ index. Four articles, with 9 arms, containing 767 participants were included in this meta-analysis. Average HFCS and sucrose usage equated to 19% of daily caloric intake. Combined data from three studies indicated that HFCS intake does not significantly change the weight (weighted mean difference (WMD): −0.29 kg, 95% CI: −1.34, 0.77, I^2^ = 0%) when compared to the sucrose group. Concordant results were found for waist circumstance, body mass index, fat mass, total cholesterol (TC), high-density lipoprotein (HDL), low-density lipoprotein (LDL), triglyceride (TG), systolic blood pressure (SBP), and diastolic blood pressure (DBP). Moreover, overall results from three studies indicated a significant increase in CRP levels (WMD: 0.27 mg/l, 95% CI: 0.02, 0.52, I^2^ = 23%) in the HFCS group compared to sucrose. In conclusion, analysis of data from the literature suggests that HFCS consumption was associated with a higher level of CRP compared to sucrose, whilst no significant changes between the two sweeteners were evident in other anthropometric and metabolic parameters.

## Introduction

Per capita consumption of sugar in the world increased sharply in the twentieth century. Although reports suggest that this consumption has now leveled off (1), it is of concern that high levels of added sugars have negative effects on human health (2). Sweeteners can be divided into two groups: nutritional and non-nutritional; where nutritional sweeteners contain monosaccharides (fructose and galactose), disaccharides (sucrose, maltose, and maize-based sweeteners) and polyols (sugar alcohol) that produce energy. On the other hand, non-nutritional sweeteners, such as acesulfame K, aspartame, neotame, saccharin and sucralose, do not contain sweet calories (3). Saccharose (GI 61-68) is a sweetener and well-used ingredient in the food industry, however, can cause harmful health problems (4). Glucose, fructose, and sucrose are natural nutrient sweeteners, where sucrose consists of a glucose molecule that is linked to a fructose molecule. High fructose corn syrup is a mixture of glucose and free fructose. The wide distribution of hexokinase enzymes, which are essential for the production of glucose-6-phosphate and glycolytic enzymes, means that glucose is metabolized by all cells of the organism. Fructose, on the other hand, is not so readily phosphorylated by the hexokinases and is metabolized exclusively in the intestine, liver and kidney. Glucose metabolism is regulated by insulin after a meal, whilst after consuming a fructose-only diet, the bulk enters the intestine and the liver, with a markedly longer transit time than glucose. Up to 20% of fructose may be stored as hepatic glycogen, and a large part is converted to LDL/VLDL (5). Furthermore, energy efficiency from fructose metabolism is lower than glucose; where at lower intake, fructose stimulates the metabolic pathway of hepatic de novo lipid production more than glucose does. However, this pathway plays only a minor role in the total excretion of fructose (6–8). Fructose is a natural monosaccharide (glycemic index, GI: 19-23), used as a sweetener in the food industry, whilst high fructose corn syrup (HFCS) is a liquid substitute for sucrose with 42 or 55% (dry) fructose, and is obtained from the hydrolysis of corn starch to glucose using glucoamylase and α-amylase, followed by glucose isomerization to fructose, which results in the production of a mixture of glucose and fructose (9, 10). HFCS can provide flavor, color, texture, stability, and freshness in some food products, such as beverages, processed foods, baking products, ice cream and confectioneries (11). It has been demonstrated in a rat study that the addition of sugars, in general, and HFCS directly or indirectly contribute to obesity, as well as various types of metabolic disorders and diseases (12). Studies have shown that excess consumption of sugar can lead to weight gain, confers a greater risk of developing metabolic heart disease, and an increased risk of early mortality (2, 13–15). HFCS is a nutritional sweetener that is thought to be harmful for human health, partly attributed to preliminary research that shows consumption of large quantities of fructose (i.e. the main constituent of HFCS) can lead to deleterious metabolic consequences in the body (16–18). Sucrose is also comprised of glucose and fructose, which is absorbed in the digestive tract (19). Therefore, there is minimal difference between HFCS and sucrose, due to the ability of the human digestive system to absorb sucrose and fructose. Previous trials have shown that the use of HFCS in comparison with sucrose yields no significant difference in health-related indicators, such as glycemic index, calorie intake, lipid metabolism and inflammation (20–23). However, there is evidence to suggest that fructose consumption in comparison with sucrose has a significantly greater effect on indicators of health (24, 25). Thus, given the current equivocality in the field, the aim of the present study is to determine the impact of HFCS vs. sucrose on anthropometric and metabolic parameters.

## Methods

This meta-analysis was performed and reported based on the ‘Preferred Reporting Items for Systematic Review and Meta-analysis’ (PRISMA) statement (26). The research questions were designed using the population, intervention, comparison, and outcomes (PICO) method.

### Search strategy

We searched PubMed, Scopus, Cochrane Central and Clinicaltrials.gov and web of sciences, from database inception to May 2022. The search strategy combined Medical Subject Heading (MeSH) and non-MeSH terms: (“Clinical Trials as Topic”[Mesh] OR “Cross-Over Studies”[Mesh] OR “Double-Blind Method”[Mesh] OR “Single-Blind Method”[Mesh] OR “Random Allocation”[Mesh] OR RCT[Title/Abstract] OR “Intervention Studies”[Title/Abstract] OR “intervention”[Title/Abstract] OR “controlled trial”[Title/Abstract] OR “randomized”[Title/Abstract] OR “randomized”[Title/Abstract] OR “random”[Title/Abstract] OR “randomly”[Title/Abstract] OR “placebo”[Title/Abstract] OR “assignment”[Title/Abstract]) AND ((“High Fructose Corn Syrup 55%”[tiab] OR “HFCS-55”[tiab] OR “High Fructose Corn Syrup”[tiab] OR “High Fructose Corn Syrup”[Mesh]) AND ((((Sucrose[Title/Abstract] OR “Sucrose”[Mesh]) OR “Sugar”[tiab]) OR “Sugars”[Mesh]) OR “Sugars”[tiab])) (see Supplementary Table 1). Two of the authors (J.R and H.K) implemented independent searches to avoid missing any relevant articles.

### Eligibility criteria

The inclusion criteria were as follows: (1) RTs (2), HFCS use vs. sucrose (3), treatment for at least 48 weeks (4), reported sufficient information on BMI, WC, and body weight, lipid profile, CRP, blood pressure, and blood glucose both in High Fructose Corn Syrup group and Sucrose administration group (5), inclusion of patients ≥ 18 y old. Publications that did not provide outcome measures at study baseline and end of the intervention (or changes in outcome measures), Observational studies, case reports, case series, non-systematic reviews and trials published as abstracts were excluded.

### Data extraction and quality assessment

Titles and abstracts were independently reviewed against eligibility criteria by two investigators (C.C and Y.Z). Any controversies or disagreements were resolved by the third author (H.K) *via* cooperative triangulation. Choice of the final eligible articles was according to the consensus between three investigators (C.C, Y.Z, and H.K). Moreover, with reference to the measures of outcome, the following data were extracted: (1) study's first author, (2) year of publication, (3) type of study population, (4) number of people in each groups, participants' mean age, participants' gender, study design, trial duration and results (means and standard deviations of outcomes at baseline, end of study and/or changes between baseline and end-intervention). To assess the quality of studies, we used the Cochrane Collaboration tool to assess risk of bias (27).

### Quantitative data synthesis

The changes in the mean values of body weight, body mass index, waist circumstance, fat mass, TC, HDL, LDL, TG, CRP, SBP, DBP was calculated for all included studies. The combined changes in outcomes were assessed as the difference (High Fructose Corn Syrup minus Sucrose) between its differences (post-trail values minus beginning trail) in the High Fructose Corn Syrup group and the Sucrose group. Standard deviations (SDs) of the mean difference were estimated using the following formula: SD = square root [(SDpre-intervention)2 C (SDpost- intervention)2 – (2R ^*^ SDpre-intervention^*^SDpost- intervention)], supposing a correlation coefficient (R) = 0.5 (28). A random-effects model and the generic inverse variance method were applied to assess overall effect size and the heterogeneity among studies. Heterogeneity analysis was performed using the Cochran Q test and the I^2^ index. Moreover, probable publication bias was determined visually by funnel plot and confirmed, statistically, through the Egger's and begg's tests. All statistical analyses were performed using Stata software, version 14 (Stata Corp. College Station, Texas, USA).

## Results

**Figure 1:**
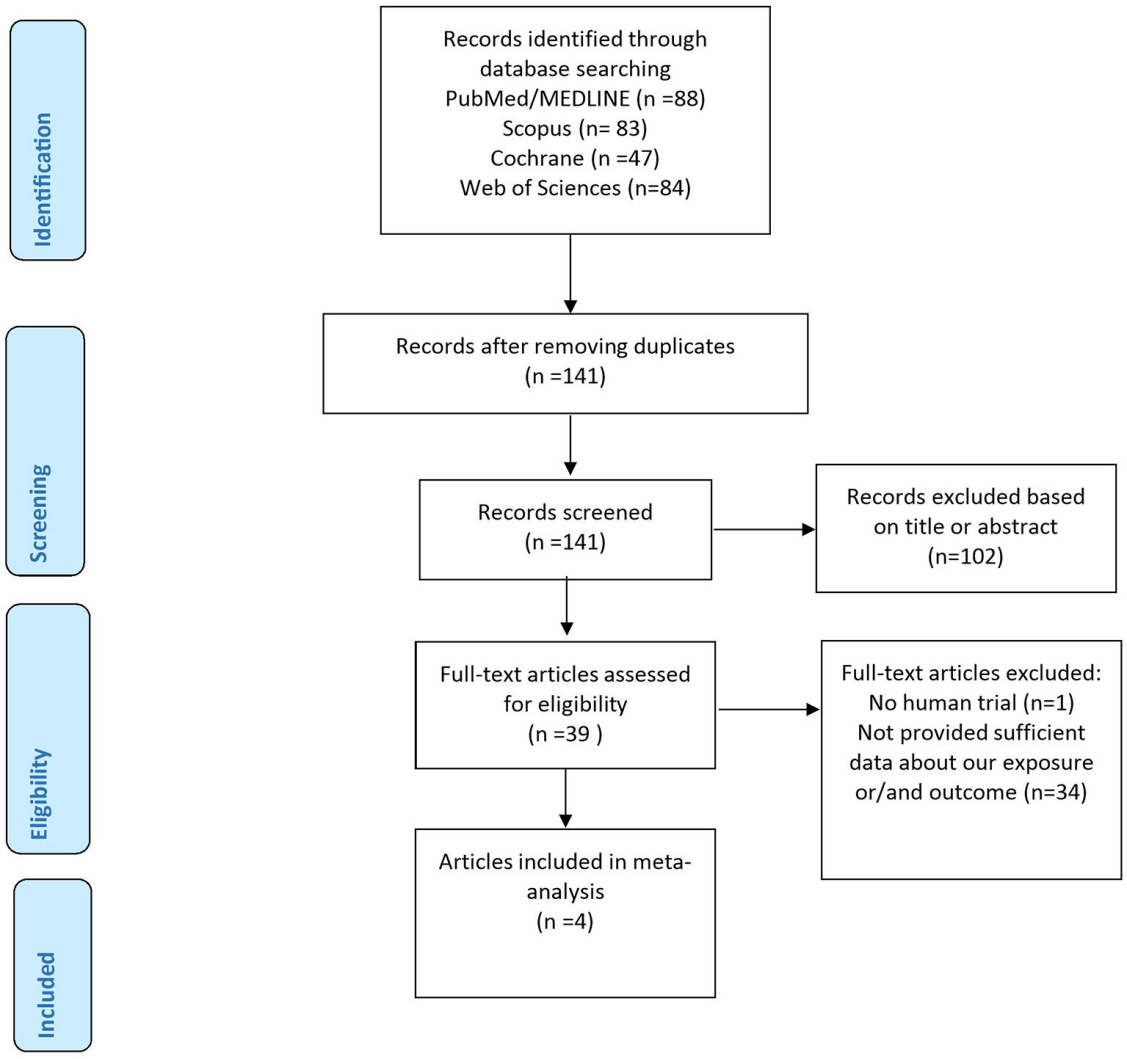
Flow chart of included studies.

Figure 1 details the flow chart of our systematic search. In our comprehensive search of PubMed, Scopus, Web of Science, and Cochrane Library, and after removing duplicates, 141 articles were included in screening, and 102 articles were excluded because they did not meet the aforementioned inclusion criteria. In full-text screening, 35 articles were removed, and four articles, with 9 arms, containing 767 participants were included in this meta-analysis (20, 29–31).

### Study characteristics and risk of bias assessment

**Figure 2:**
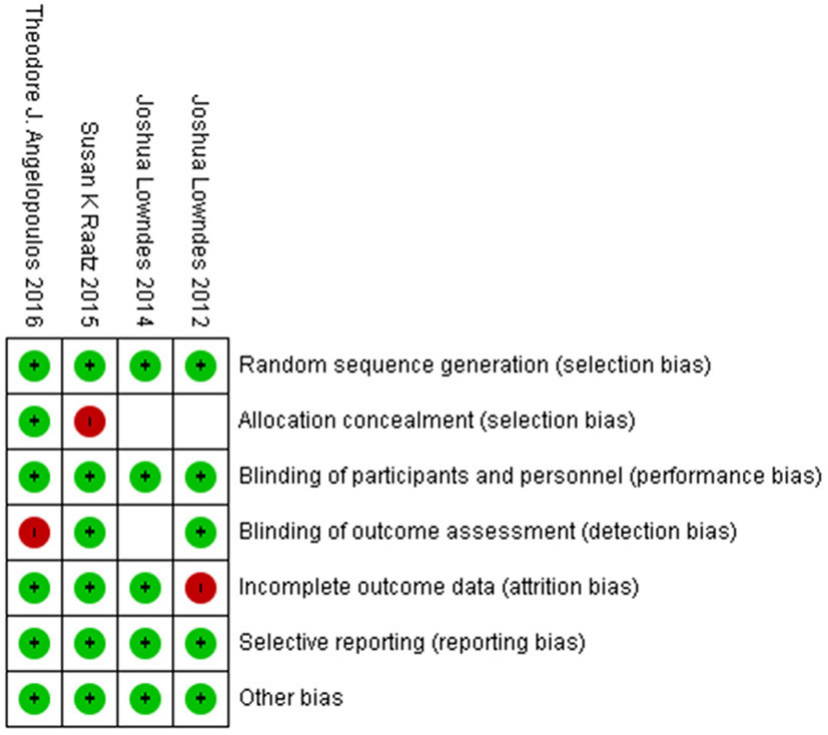
Risk of bias assessment of included studies.

Characteristics of the included studies are provided in Table 1. Mean age of participants was 41.51 years, ranging from 37 to 52 years and mean length of the interventions was 8.5 weeks, ranging from 2 to 12 weeks. All studies were conducted on both genders in the USA and articles were published between 2012 and 2016. Risk of bias of included studies was evaluated by the Cochrane's Handbook checklist, and most papers were confirmed as having good quality (Figure 2) (20, 29–31).

**Table 1:** Characteristics of included studies.

**Author**	**Country (year)**	**Study design (duration)**	**Gender**	**Age (year)**	**Patient features**	**Sample size HFCS/sucrose**	**Dose (mg)**	**Outcome(s)**
Theodore J. Angelopoulos	USA (2016)	Parallel (10W)	Both	37.66	Healthy	91/89	18%	Body weight, WC, BMI, SBP, DBP, Glucose, TG, LDL, HDL, TC, CRP
Susan K Raatz	USA (2015)	Crossover (2W)	Both	38.9	Normal glucose tolerance (GT)	28/28	50 g	Body weight, BMI, SBP, DBP, Glucose, TG, LDL, HDL, TC, CRP
Susan K Raatz	USA (2015)	Crossover (2W)	Both	52.1	Impaired glucose tolerance (IGT)	27/27	50 g	Body weight, BMI, SBP, DBP, Glucose, TG, LDL, HDL, TC, CRP
Joshua Lowndes	USA (2014)	Parallel (10W)	Both	40.19	Overweight or obese individuals	51/53	30%	Body weight, WC, BMI, fat mass, SBP, DBP, Glucose, TG, LDL, HDL, TC, CRP
Joshua Lowndes	USA (2014)	Parallel (10W)	Both	40.19	Overweight or obese individuals	60/64	18%	Body weight, WC, fat mass, SBP, DBP, Glucose, TG, LDL, HDL, TC, CRP
Joshua Lowndes	USA (2014)	Parallel (10W)	Both	40.19	Overweight or obese individuals	69/58	8%	Body weight, WC, BMI, fat mass, SBP, DBP, Glucose, TG, LDL, HDL, TC, CRP
Joshua Lowndes	USA (2012)	Parallel (12W)	Both	40	Overweight/obese participants	36/29	10%	BMI
Joshua Lowndes	USA (2012)	Parallel (12W)	Both	42.9	Overweight/obese participants	24/33	30%	BMI

### Meta-analysis results

**Figure 3:**
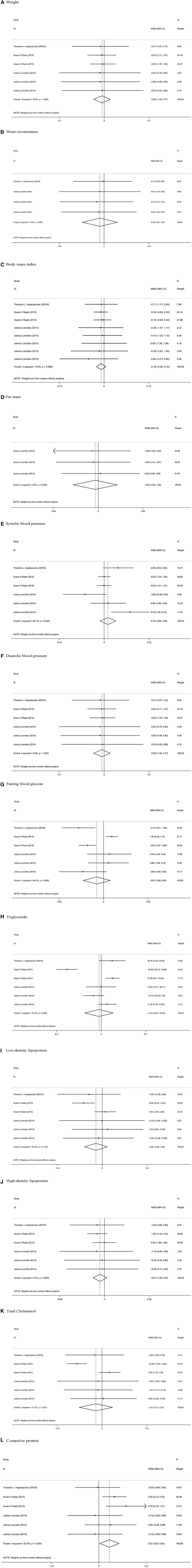
Meta-analysis of effect of High-fructose corn syrup on. **(A)** Weight. **(B)** Waist circumstance. **(C)** Body mass index. **(D)** Fat mass. **(E)** Systolic blood pressure. **(F)** Diastolic blood pressure. **(G)** Fasting blood glucose. **(H)** Triglyceride. **(I)** Low-density lipoprotein. **(J)** High-density lipoprotein. **(K)** Total Cholesterol. **(L)** C-reactive protein.

Three studies, with six arms, including 326 participants in intervention groups and 319 participants in control groups reported weight as an outcome measure (20, 29, 30), and combined results did not show any significant change in HFCS (WMD: −0.29 kg, 95% CI: −1.34, 0.77, I^2^ = 0%) compared to the sucrose group (Figure 3). There were comparable results for waist circumstance (WMD: −0.39 cm, 95% CI: −2.04, 1.25, I^2^ = 0%) (29, 30), Body mass index (WMD: −0.18 kg/m^2^, 95% CI: −0.46, 0.10, I^2^ = 0%) (20, 29–31), and fat mass (WMD: −0.24 %, 95% CI: −2.04, 1.55, I^2^ = 0%) (30).

Among eligible studies, three trails, with six arms, containing a total of 645 participants (326 participants in HFCS group and 319 participants in sucrose group) reported SBP, DBP, fasting blood glucose (FBG), TG, LDL, HDL, and TC as an outcome measure (20, 29, 30). Pooled results from the random-effects model did not show any significant change between the two groups in SBP (WMD: 0.76 mmHg, 95% CI: −0.86, 2.38, I^2^ = 62%), DBP (WMD: −0.29 mmHg, 95% CI: −1.34, 0.77, I^2^ = 0%), FBG (WMD: −0.87 mg/dl, 95% CI: −2.56, 0.82, I^2^ = 84%), TG (WMD: −4.15 mg/dl, 95% CI: −30.37, 22.07, I^2^ = 91%), LDL (WMD: −2.02 mg/dl, 95% CI: −5.69, 1.65, I^2^ = 42%), HDL (WMD: −0.61 mg/dl, 95% CI: −1.55, 0.32, I^2^ = 0%), and TC (WMD: −2.45 mg/dl, 95% CI: −9.12, 4.22, I^2^ = 74%).

Overall results from three studies indicated a significant increase in CRP levels (WMD: 0.27 mg/l, 95% CI: 0.02, 0.52, I^2^ = 23%) in the HFCS group compared to the sucrose group (20, 29, 30).

### Publication bias

Funnel plot assessment did not show any asymmetry in included studies (Supplemental Figure 1). Furthermore, The Egger's and Begg's tests did not show any significant publication bias for Weight (*p* = 0.09 and *p* = 0.57), WC (*p* = 0.25 and *p* = 0.99), BMI (*p* = 0.43 and *p* = 0.32), Fat mass (*p* = 0.62 and *p* = 0.60), SBP (*p* = 0.51 and *p* = 0.57), DBP (*p* = 0.60 and *p* = 0.57), FBS (*p* = 0.39 and *p* = 0.85), TG (*p* = 0.50 and *p* = 0.57), LDL (*p* = 0.84 and *p* = 0.57), HDL (*p* = 0.96 and *p* = 0.57), TC (*p* = 0.69 and *p* = 0.85), and CRP (*p* = 0.23 and *p* = 0.34), respectively.

## Discussion

Numerous empirical evidence has indicated that sugars, particularly HFCS and sucrose, can affect various anthropometric and metabolic parameters (6, 12). However, it remains debatable whether the effects of HFCS and sucrose are of equal magnitude. Some studies have demonstrated that consumption of HFCS and sucrose elicited comparable effects, while some other reports noted a marked difference between the two sugars (20, 22, 25). In this work, we performed a meta-analysis to determine whether the effect of HFCS and sucrose on anthropometric and metabolic parameters were concordant. We found that HFCS was significantly associated with an increased CRP level, compared to sucrose. However, we observed no difference between HFCS and sucrose in terms of their effects on weight, WC, BMI, fat mass, SBP, DBP, FBS, TG, LDL, HDL, and TC.

CRP is a biomarker for inflammation; and several previous investigations have shown that fructose-containing sweeteners, such as HFCS and sucrose, can induce the inflammatory process (32, 33). This is conceivably attributable to the unique metabolic process of fructose, which can cause oxidative stress to cells by elevating the intracellular levels of uric acid and reactive oxygen species (33, 34). To overcome the oxidative stress, cells release molecules such as monocyte chemotactic protein 1 (MCP-1), tumor necrosis factor (TNF) and interleukins, which are pro-inflammatory in nature and thus augment the inflammation process (33). HFCS and sucrose both contain the fructose moiety; however, the content of fructose in HFCS and sucrose is different, which conceivably explains why the two sugars induced inflammation differently. Despite this, in the present study, no significant difference was observed for any other anthropometric or metabolic parameter. The lack of significant difference could be attributed to several reasons. First, in some of the included studies, the duration of the experiment was short. For example, in the study by Raatz et al., the participants consumed the sugars for only 2 weeks (20); this duration is likely insufficient for significant weight changes to be manifest. In addition, compliance with the prescribed sugars was generally obtained through self-reported information from the participants, which could cause validity and bias issues (35, 36). Moreover, the studies generally delivered the sugars through milk or other foods, which contained other nutrients, such as vitamin D and calcium. These nutrients can affect several metabolic parameters, for example, vitamin D has been shown to alter cholesterol levels, especially LDL (37–39). On the other hand, calcium supplementation is known to decrease blood pressure (40). The presence of these nutrients in the foods used to deliver the sugars could act as confounders for our analyses; therefore, the findings should be interpreted with caution. Notwithstanding, the amount/concentration of the sugars consumed were different across the included studies; hence, it is conceivable that the effects elicited by the sugars was masked by studies which examined the lower concentration of the sugars.

There are a number of strengths and limitations of the present study. Firstly, through the amalgamation of studies, we yielded an increased statistical power of analysis compared to the individual studies (41). In addition, most studies included in the present meta-analysis had high methodological quality and employed a randomized trial design. A randomized trial minimizes various types of biases and confounders and produces strong empirical evidence of the effects of an intervention (42). It is also noteworthy that publication bias was not evident in our meta-analysis, which suggests that the results obtained are likely not exaggerated (43). Apart from that, the sugars consumed by the participants were incorporated into their normal diets, which allowed a more accurate evaluation of the real-world effects of the added sugar compared to a totally controlled trial (20). However, one of the limitations of this meta-analysis was that subgroup analyses were not performed due to the lack of incumbent data in the included studies, which was out of the operational control of the study. For instance, the effects of HFCS and sucrose on male and female participants were not evaluated separately in the included studies. Importantly, many human and animal studies have demonstrated that males and females have different responses to fructose (44–48); for example, triglyceride levels in young women are less sensitive to modifications by fructose compared to men (44, 45, 48), and therefore represents a pragmatic avenue for further research. Besides gender, subgroup analysis by people of different age groups was not performed, although animal studies have indicated that fructose can induce metabolic changes differently in young and adult rats (49). Another limitation of this study was that, as mentioned above, compliance with the assigned sugar intake in the included studies was generally dependent on the self-reported information from the participants. This can cause misleading results if the participants did not adhere to the prescribed diet (35, 36). Finally, in this meta-analysis, we were unable to correct for some potential confounding factors, such as the co-incorporation of added vitamin D and calcium in the food used to deliver the sugars.

## Conclusion

In conclusion, analysis of data from the literature suggests that HFCS is associated with a higher level of CRP compared to sucrose, and that little differences exist in other anthropometric and metabolic parameters. Nonetheless, considering the limitations of the study, careful interpretation of the results is warranted. Future studies are needed to address the limitations above, particularly related to gender-mediated differences. However, the present work successfully provided a reliable estimate of the effect and difference of HFCS and sucrose in a number of anthropometric and metabolic parameters.

## Data availability statement

The original contributions presented in the study are included in the article/Supplementary materials, further inquiries can be directed to the corresponding author.

## Author contributions

XL, YunqL, YuejL, YunpL, and HK designed the study. The literature search, screening data, and data extraction were done by SY, GW, XC, and YucaL. Quality assessment was carried out by YunpL and HK. XL and YunqL analyzed and interpreted data. XL, YunqL, YuejL, YunpL, and HK wrote and edited the manuscript. All authors read and approved the final manuscript.

## Conflict of interest

Authors YucaL and HK were employed by Lairui Biotechnology (Yunnan) Co., Ltd. The remaining authors declare that the research was conducted in the absence of any commercial or financial relationships that could be construed as a potential conflict of interest.

## Publisher's note

All claims expressed in this article are solely those of the authors and do not necessarily represent those of their affiliated organizations, or those of the publisher, the editors and the reviewers. Any product that may be evaluated in this article, or claim that may be made by its manufacturer, is not guaranteed or endorsed by the publisher.
