# Supplementary material for: The effect of high-fructose corn syrup vs. sucrose on anthropometric and metabolic parameters: A systematic review and meta-analysis
Source: Front Nutr. 2022 Sep 27;9:1013310. doi: 10.3389/fnut.2022.1013310 (PMC9551185; doi:10.3389/fnut.2022.1013310)
Supplement: Supplementary file 1 [file Table_1.pdf]

Supplementary table 1. Search strategy.

PubMed/MEDLINE	Scopus	Cochrane Library	Web of Science
("Clinical Trials as Topic"[Mesh] OR "Cross-Over Studies"[Mesh] OR "Double-Blind Method"[Mesh] OR "Single-Blind Method"[Mesh] OR "Random Allocation"[Mesh] OR RCT[Title/Abstract] OR "Intervention Studies"[Title/Abstract] OR "intervention"[Title/Abstract] OR "controlled trial"[Title/Abstract] OR "randomized"[Title/Abstract] OR "randomised"[Title/Abstract] OR "random"[Title/Abstract] OR "randomly"[Title/Abstract] OR "placebo"[Title/Abstract] OR "assignment"[Title/Abstract]) AND (("High Fructose Corn Syrup 55%"[tiab] OR "HFCS-55"[tiab] OR "High Fructose Corn Syrup"[tiab] OR "High Fructose Corn Syrup"[Mesh]) AND ((((Sucrose[Title/Abstract] OR "Sucrose"[Mesh]) OR "Sugar"[tiab]) OR "Sugars"[Mesh]) OR "Sugars"[tiab]))	((TITLE-ABS-KEY (rct) OR TITLE-ABS-KEY ("Intervention Studies") OR TITLE-ABS-KEY (intervention) OR TITLE-ABS-KEY ("controlled trial") OR TITLE-ABS-KEY (randomized) OR TITLE-ABS-KEY (randomised) OR TITLE-ABS-KEY (random) OR TITLE-ABS-KEY (randomly) OR TITLE-ABS-KEY (placebo) OR TITLE-ABS-KEY (assignment))) AND ((TITLE-ABS-KEY ("High Fructose Corn Syrup 55%") OR TITLE-ABS-KEY ("HFCS-55") OR TITLE-ABS-KEY ("High Fructose Corn Syrup"))) AND ((TITLE-ABS-KEY (sucrose) OR TITLE-ABS-KEY (sugar) OR TITLE-ABS-KEY (sugars)))	(RCT OR "Intervention Studies" OR intervention OR "controlled trial" OR randomized OR randomised OR random OR randomly OR placebo OR assignment) AND ("High Fructose Corn Syrup 55%" OR "HFCS-55" OR "High Fructose Corn Syrup") AND (Sucrose OR Sugar OR Sugars)	TS=(RCT OR "Intervention Studies" OR intervention OR "controlled trial" OR randomized OR randomised OR random OR randomly OR placebo OR assignment) AND TS=("High Fructose Corn Syrup 55%" OR "HFCS-55" OR "High Fructose Corn Syrup") AND TS=(Sucrose OR Sugar OR Sugars)
