# Supplementary material for: The effect of high-fructose corn syrup vs. sucrose on anthropometric and metabolic parameters: A systematic review and meta-analysis
Source: Front Nutr. 2022 Sep 27;9:1013310. doi: 10.3389/fnut.2022.1013310 (PMC9551185; doi:10.3389/fnut.2022.1013310)

Supplementary Figure 1. Funnel plot to assess publication bias.

a) Weight

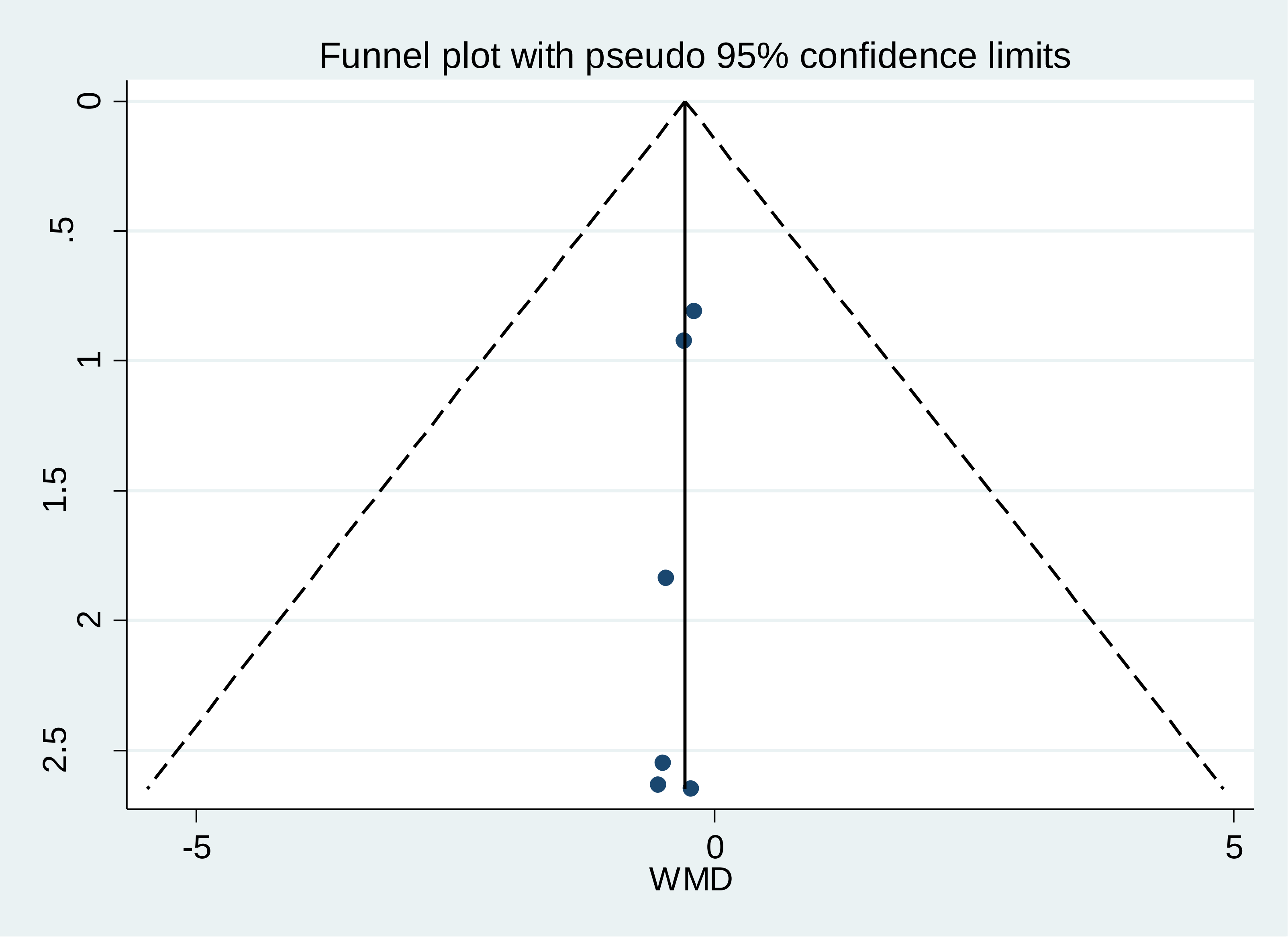

b) Waist circumstance

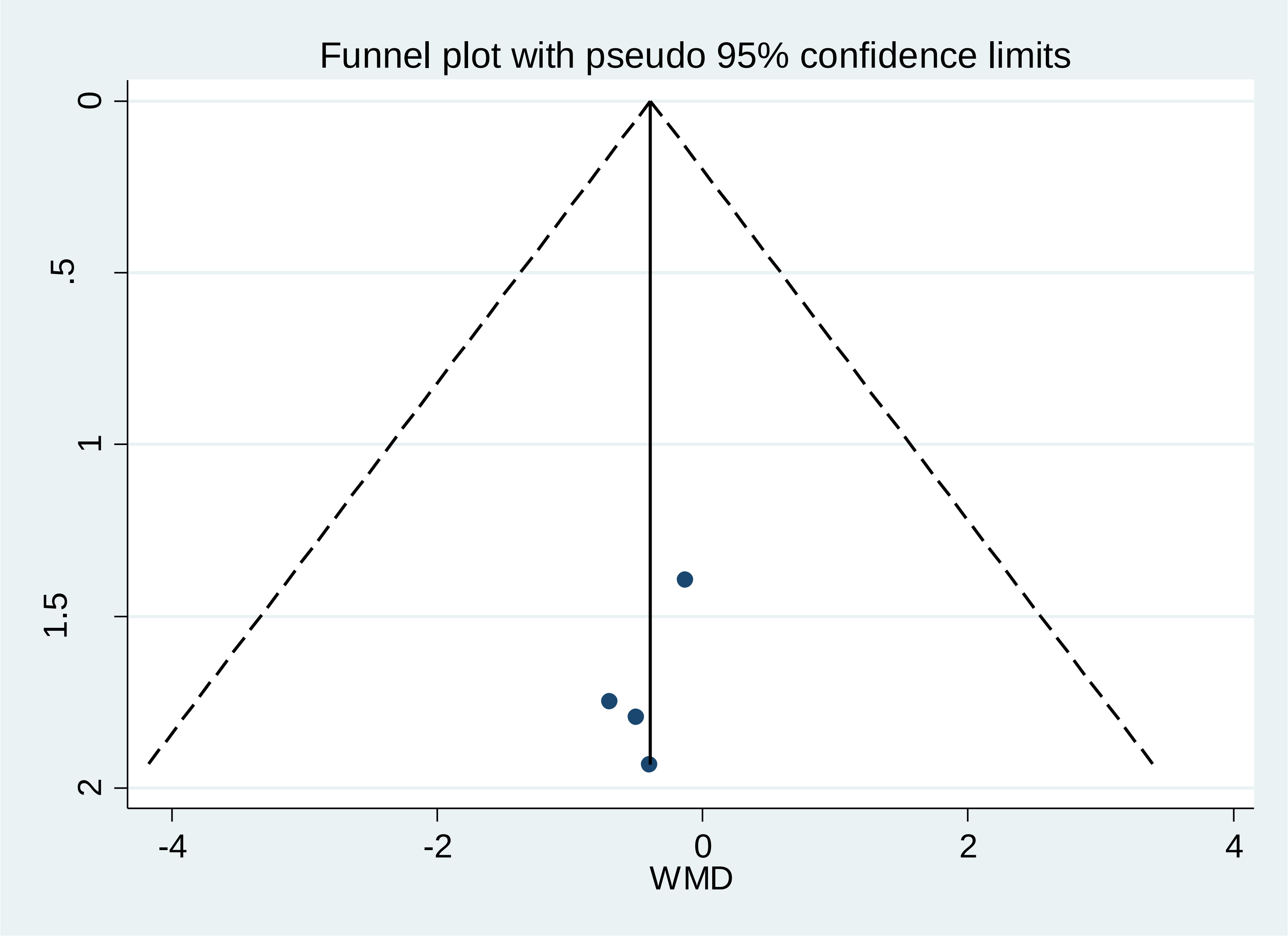

c) Body mass index

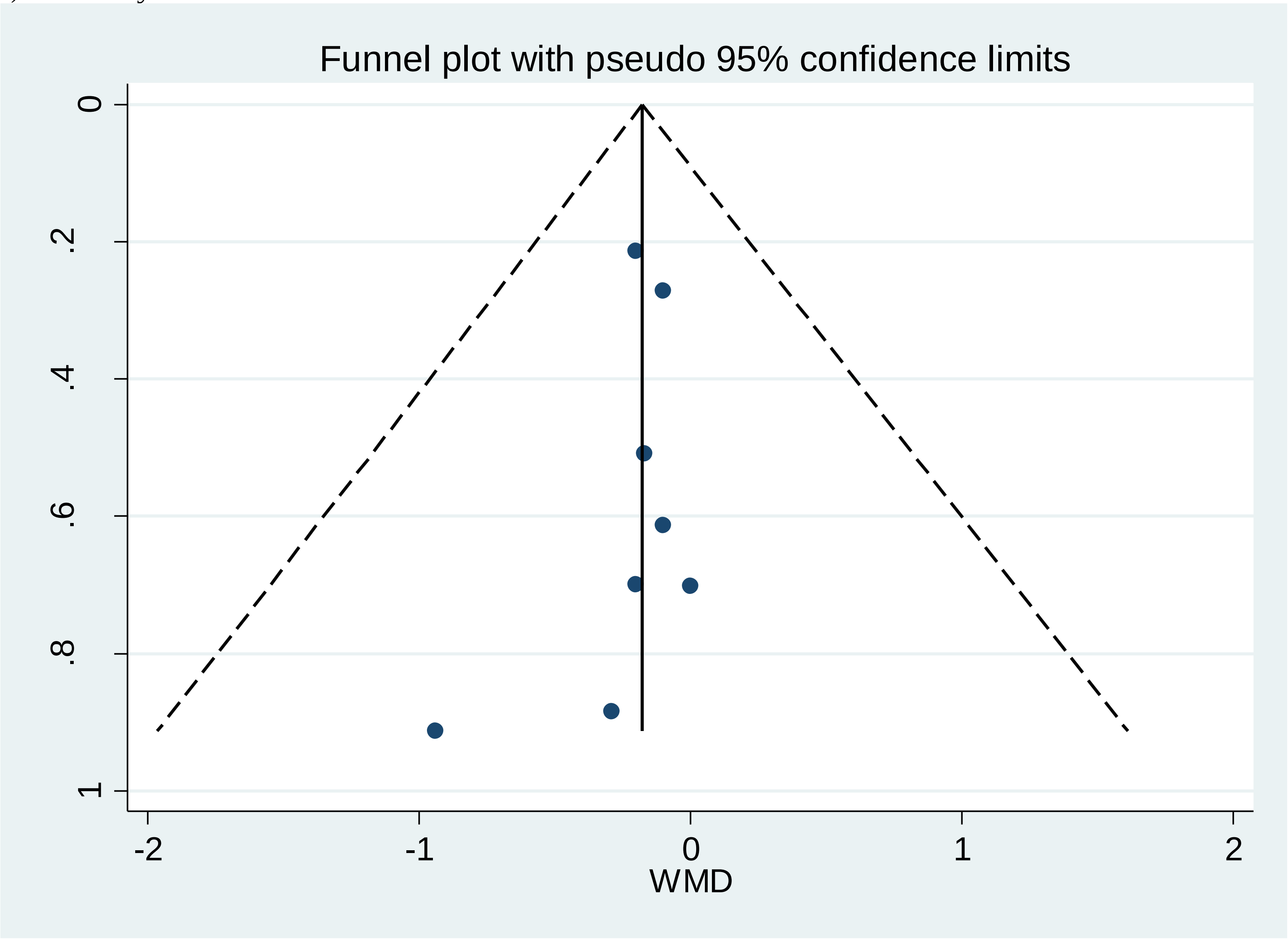

d) Fat mass

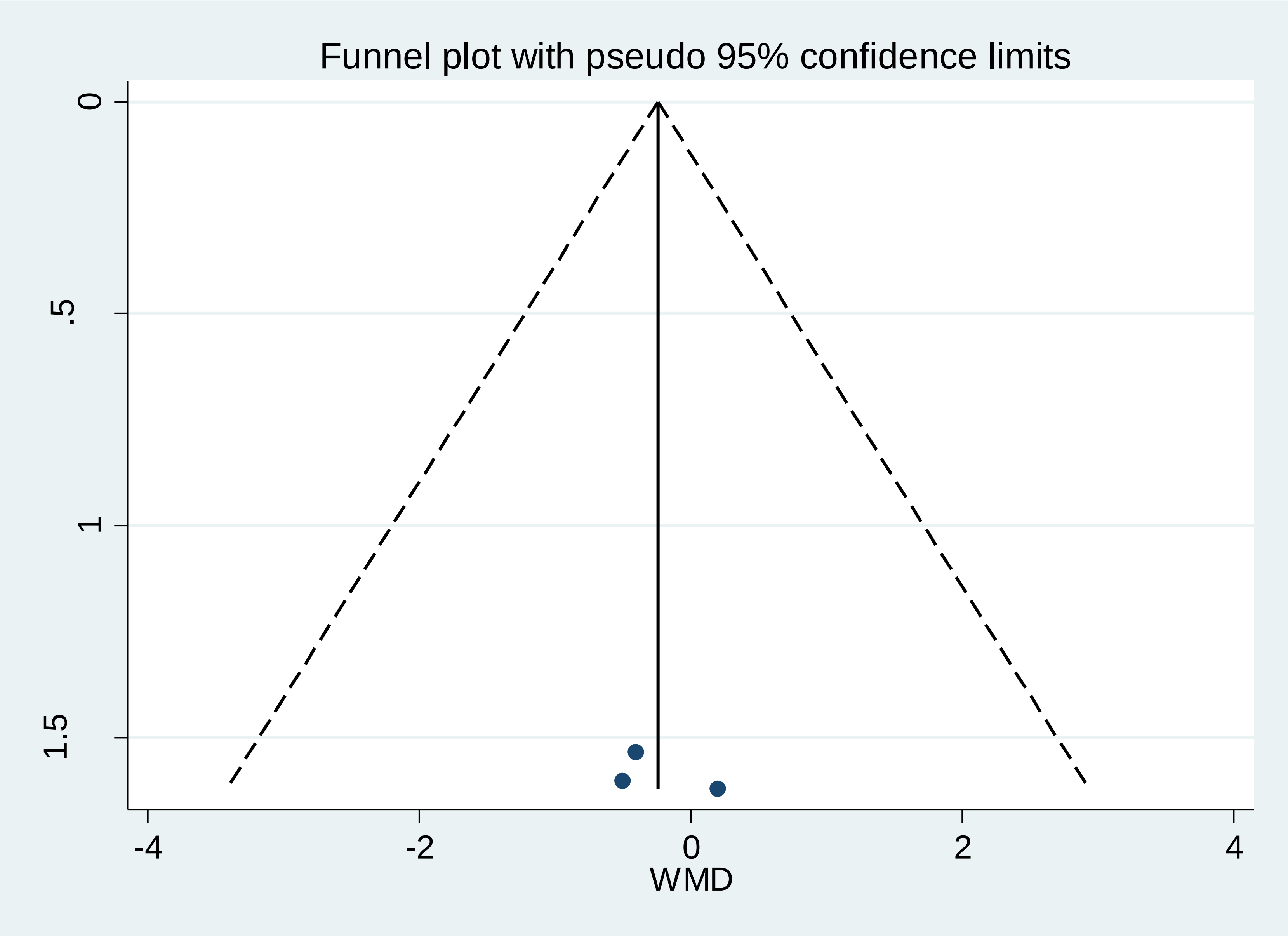
Funnel plot with pseudo 95% confidence limits
0
.5
1
1.5
-4
-2
0
2
4
WMD

e) Systolic blood pressure

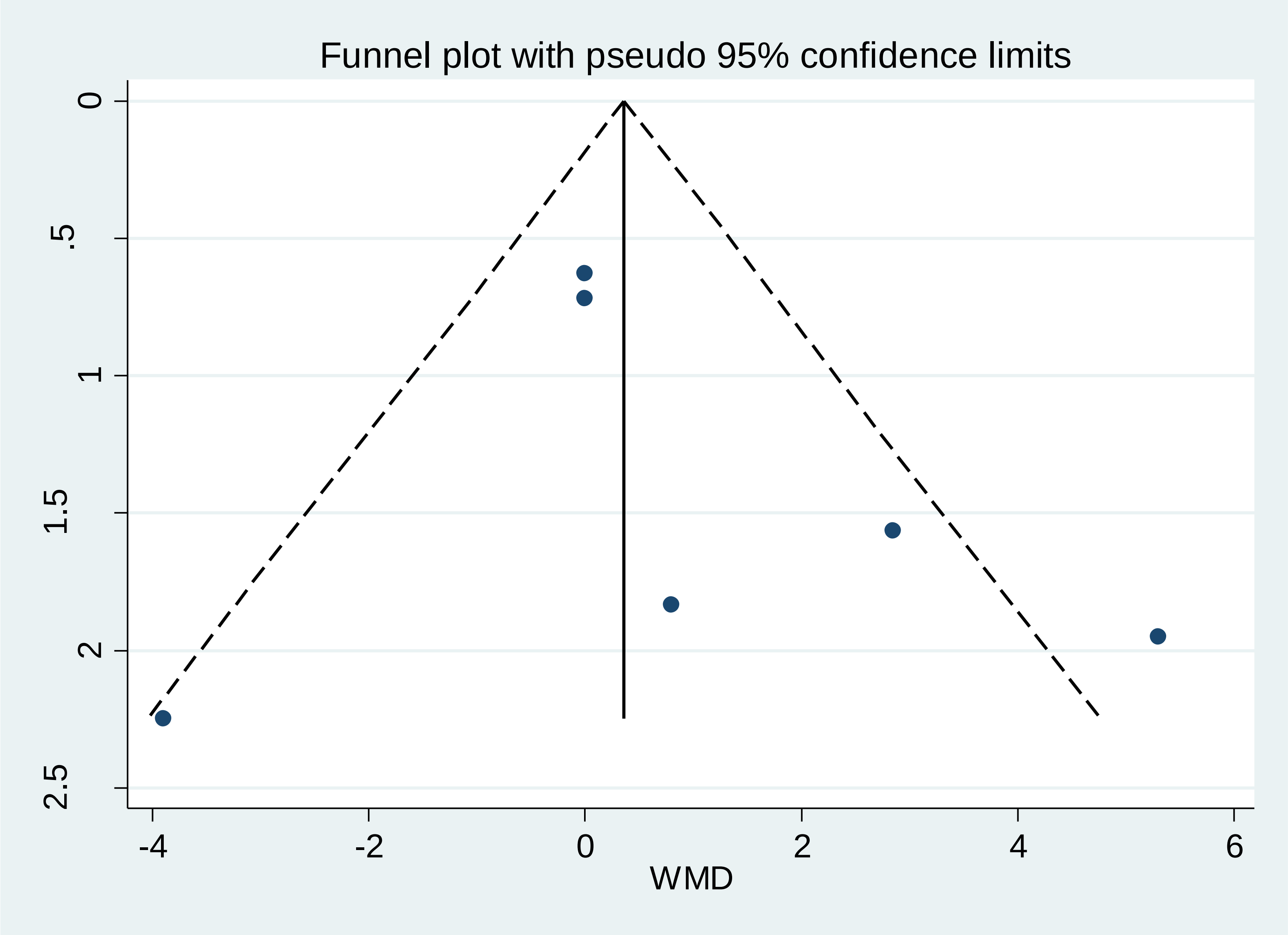

f) Diastolic blood pressure

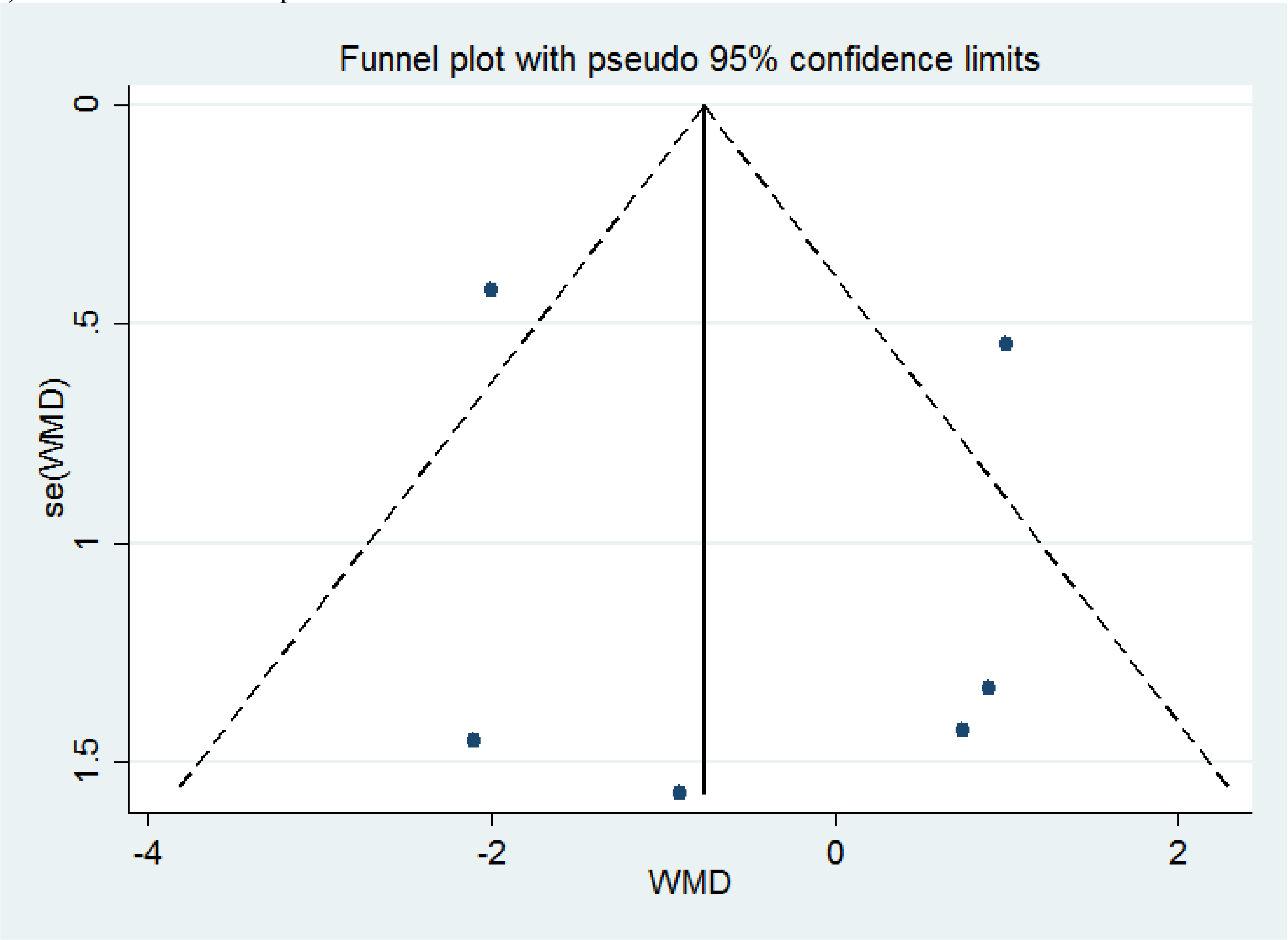

g) Fasting blood glucose

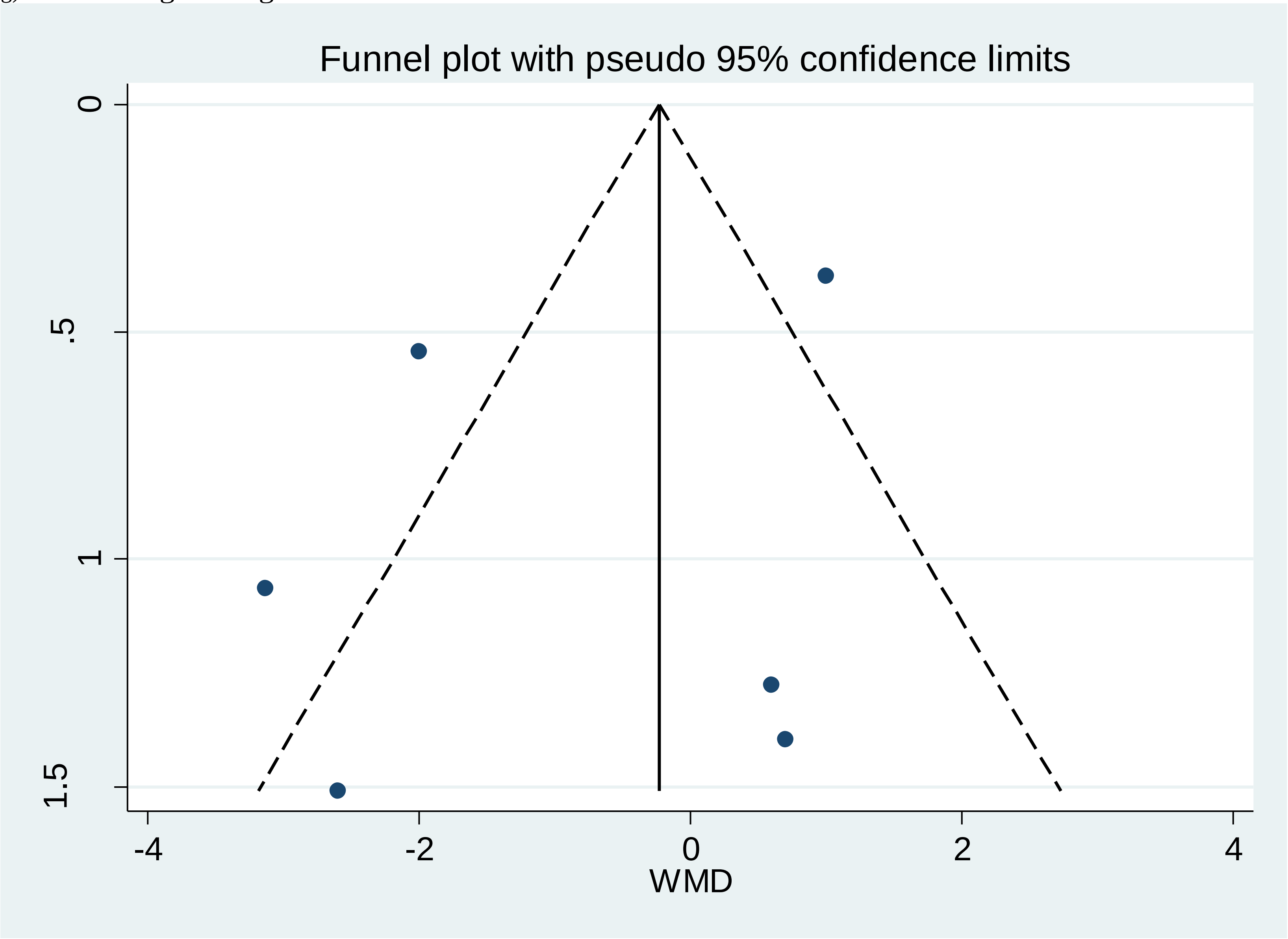

h) Triglyceride

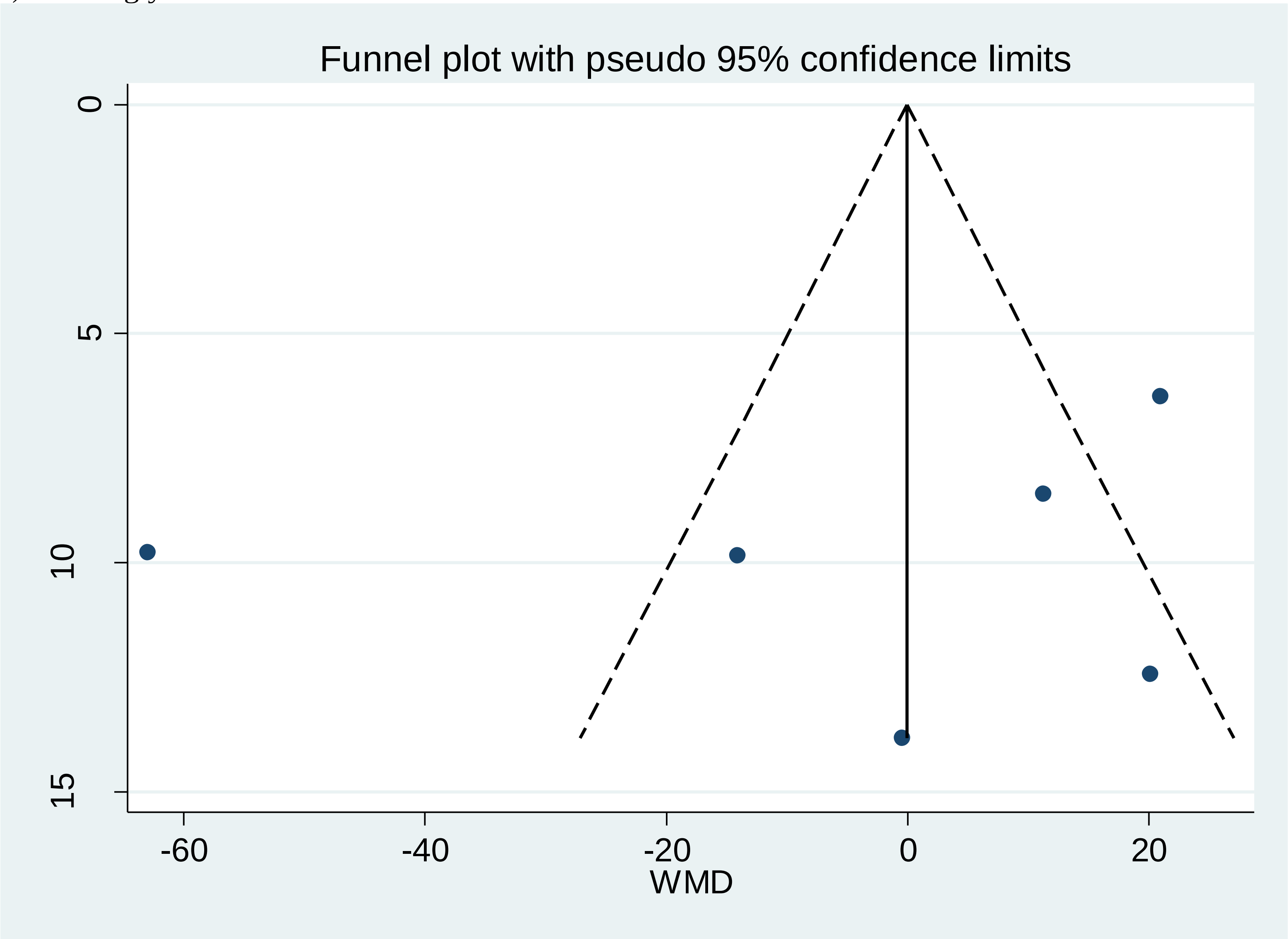

i) Low-density lipoprotein

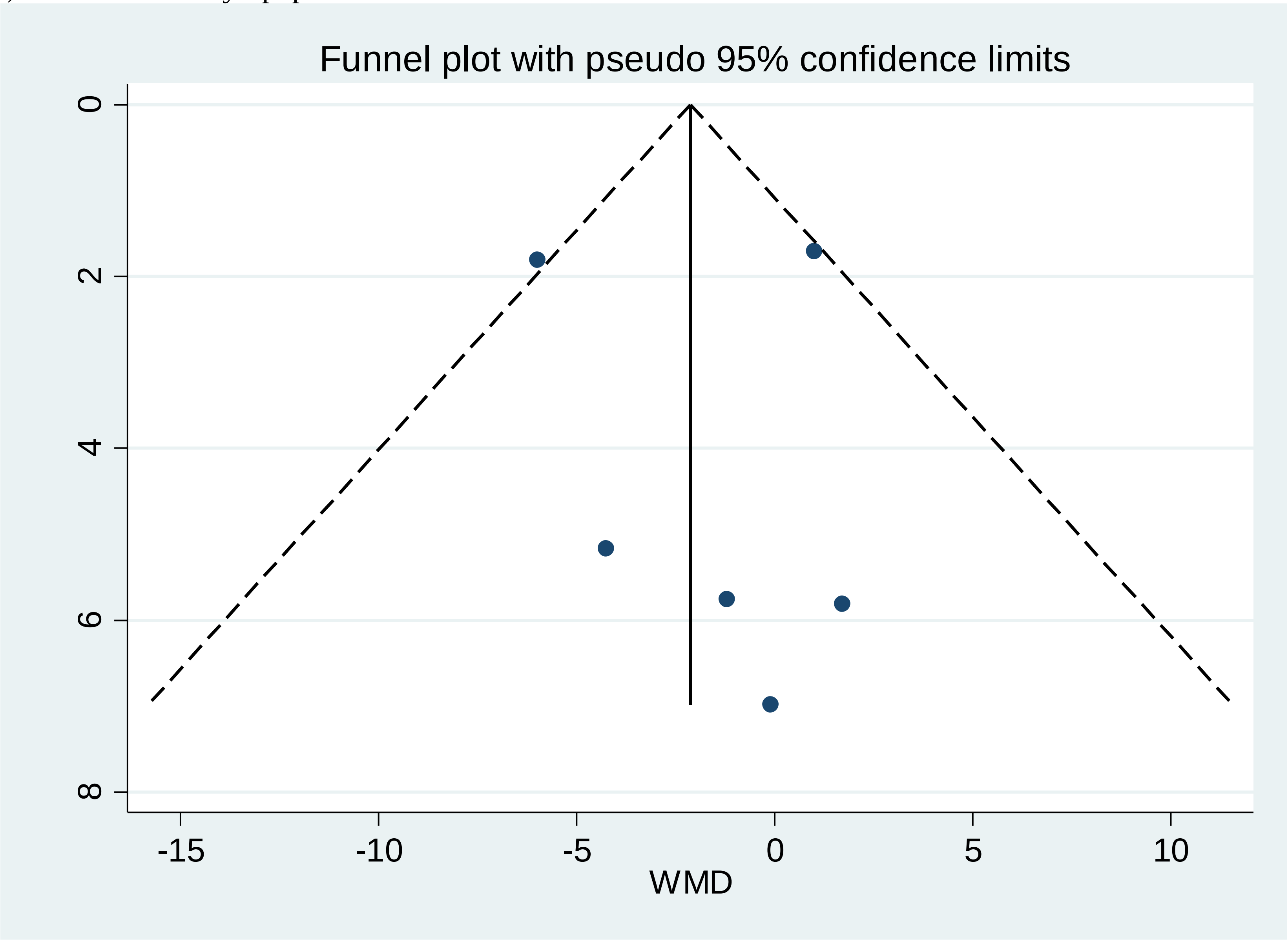

j) High-density lipoprotein

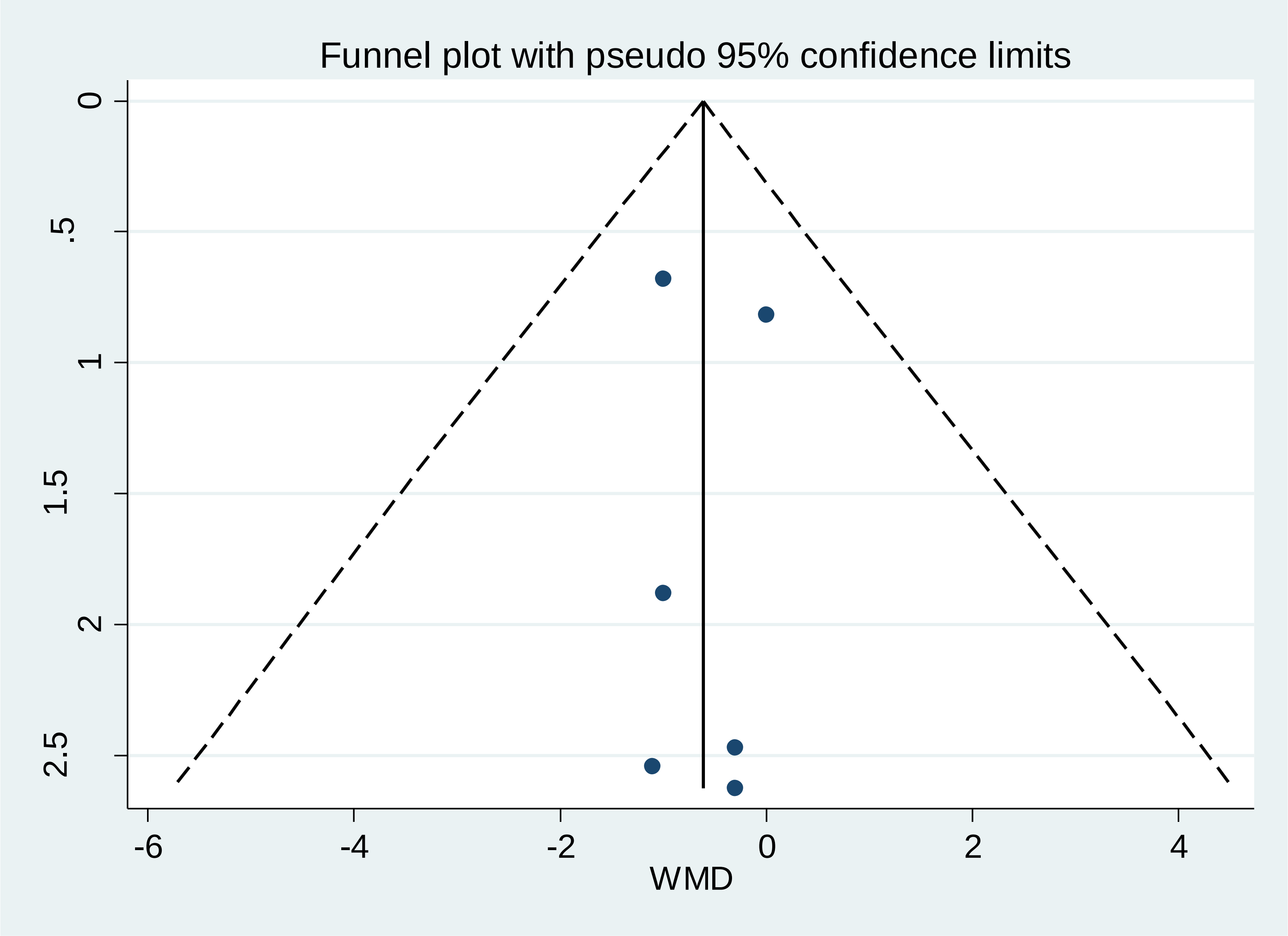

k) Total Cholesterol

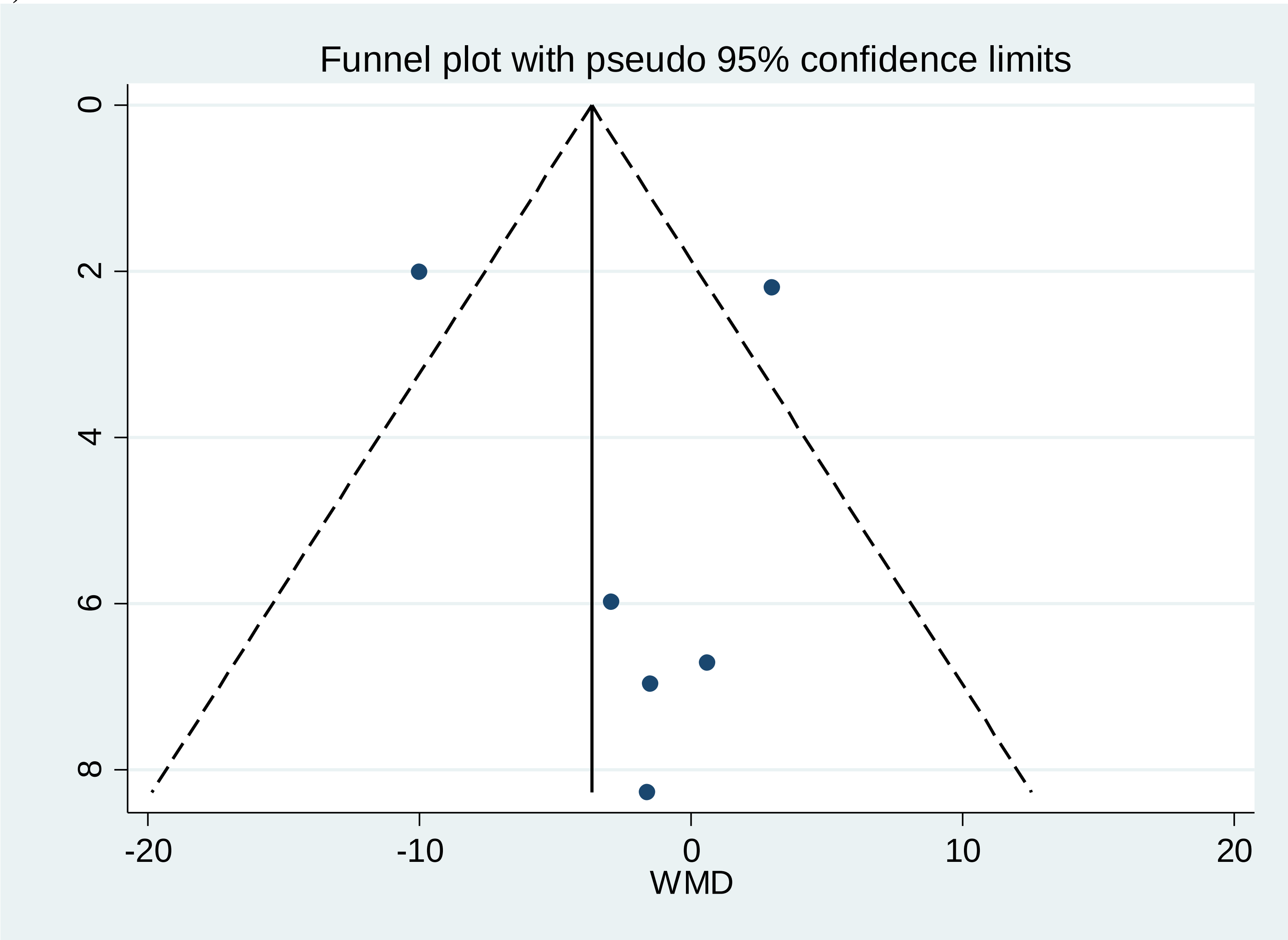

l) C-reactive protein

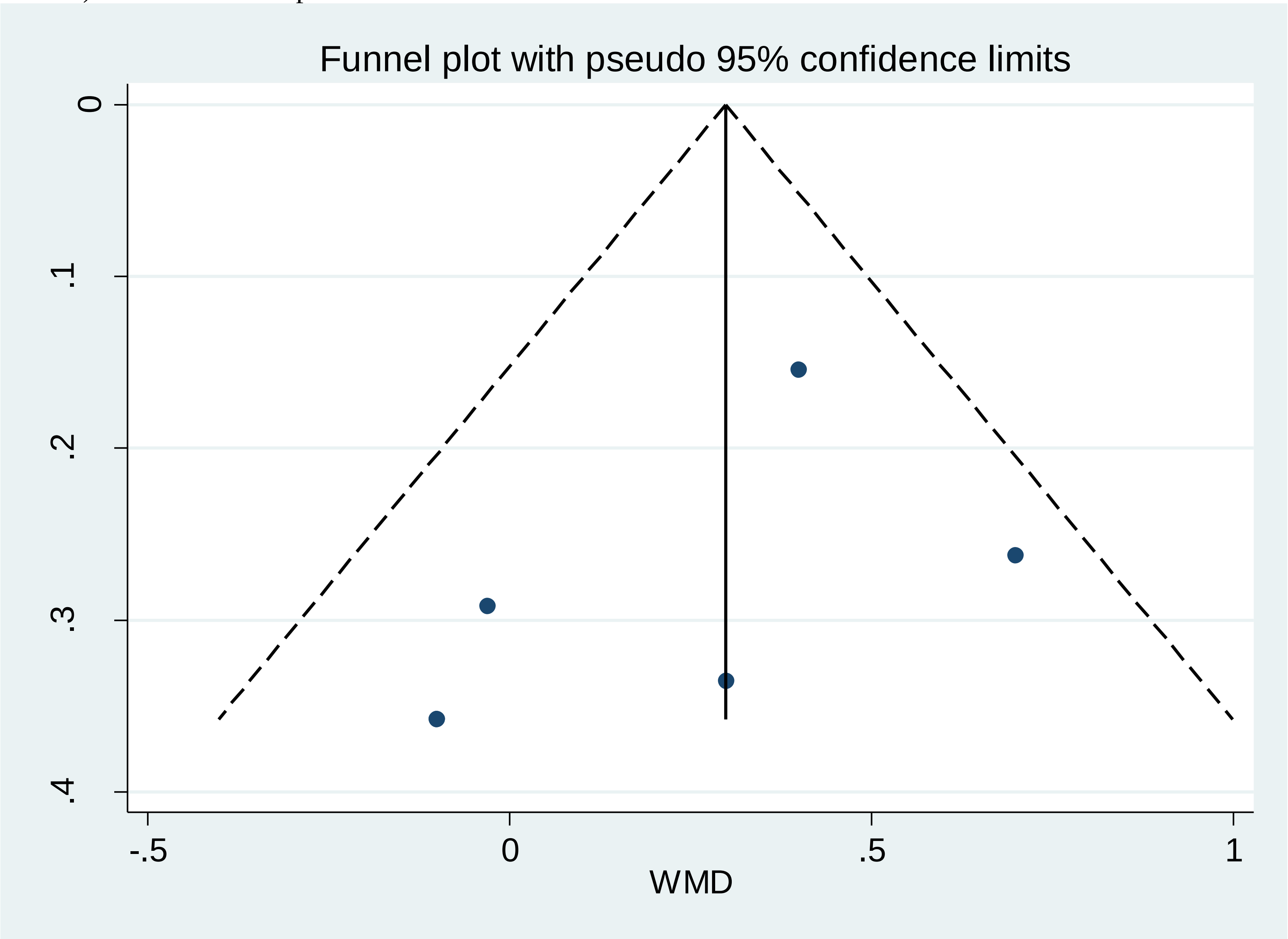

Supplement: Supplementary file 2 [file Image_1.pdf]
